# The landscape of abiotic and biotic stress-responsive splice variants with deep RNA-seq datasets in hot pepper

**DOI:** 10.1038/s41597-024-03239-7

**Published:** 2024-04-13

**Authors:** Nayoung Kim, Junesung Lee, Seon-In Yeom, Nam-Jun Kang, Won-Hee Kang

**Affiliations:** 1 Plant Systems Engineering Research Center, Korea Research Institute of Bioscience and Biotechnology (KRIBB), Daejeon, South Korea; 2 Division of Applied Life Science (BK21 Four), Gyeongsang National University, Jinju, South Korea; 3 Department of Horticulture, Institute of Agriculture & Life Science, Gyeongsang National University, Jinju, South Korea

**Keywords:** Plant stress responses, Functional clustering, Plant immunity

## Abstract

Alternative splicing (AS) is a widely observed phenomenon in eukaryotes that plays a critical role in development and stress responses. In plants, the large number of RNA-seq datasets in response to different environmental stressors can provide clues for identification of condition-specific and/or common AS variants for preferred agronomic traits. We report RNA-seq datasets (350.7 Gb) from *Capsicum annuum* inoculated with one of three bacteria, one virus, or one oomycete and obtained additional existing transcriptome datasets. In this study, we investigated the landscape of AS in response to environmental stressors, signaling molecules, and tissues from 425 total samples comprising 841.49 Gb. In addition, we identified genes that undergo AS under specific and shared stress conditions to obtain potential genes that may be involved in enhancing tolerance to stressors. We uncovered 1,642,007 AS events and identified 4,354 differential alternative splicing genes related to environmental stressors, tissues, and signaling molecules. This information and approach provide useful data for basic-research focused on enhancing tolerance to environmental stressors in hot pepper or establishing breeding programs.

## Background & Summary

Alternative splicing (AS) is a common regulatory process in eukaryotes that enables the generation of multiple mRNA isoforms from a single pre-mRNA through the utilization of distinct splicing sites^1^. This fundamental process augments the diversity of both transcriptomes and proteomes and plays a crucial role in the regulation during plant development and in response to various stressors^2^. In humans, AS occurs in over 95% of genes containing introns^3^, whereas plants exhibit a high proportion of AS events—approximately 70%—among intron-containing genes^4,5^. AS events are usually divided into five basic types^6^ dependent on their architecture: exon skipping (SE), intron retention (RI), mutually exclusive exons (MXE), alternative 3′ splice site (A3SS), and alternative 5′ splice site (A5SS).

Crops are exposed to diverse environmental stressors during their growth. In recent years, the swift progression of climate change has not only exacerbated the effects of single stressors, such as high temperature or drought, it has also produced complex stressors, such as drought accompanied by high temperature and salt damage. Consequently, crop yields have diminished by up to 80%^7^. This growing threat to crop stability has highlighted the importance of studies focused on assessing the interactions between crops and environmental stressors, revealing the need for those related to complex and single stressors.

As one of the most important crops, chili peppers (*Capsicum* spp.) are widely used as a spice or seasoning^8^. Critically, although high-quality genomic information and established gene models are available for this organism^9,10^, related research on expression of various AS isoforms is insufficient. Many high-throughput RNA sequencing (RNA-seq) datasets for *Capsicum* spp. have also been generated and analyzed^11–14^, but there is little information on AS within different tissues and in response to environmental stressors and signaling molecules. Moreover, despite an abundance of studies investigating individual stress responses or specific AS events^15–17^, the current literature notably lacks comprehensive examinations of AS events that are common across multiple stress conditions in the pepper.

**Fig. 1:**
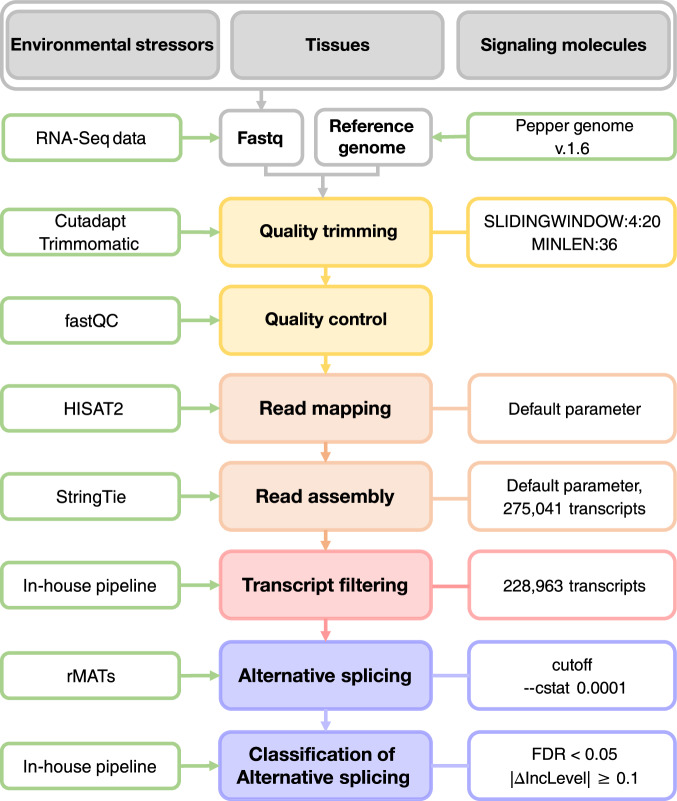
Schematic overview of our analysis pipeline. The workflow for this study includes the analysis procedures, programs, and parameters used. Detailed methodologies are described in the Methods sections.

Here, we collected RNA-seq datasets from *Capsicum annuum* (*C. annuum*) inoculated with bacteria, virus, or oomycete and identified AS events within different tissues and in response to environmental stressors and signaling molecules, via comparative differential AS analysis against large existing RNA-seq datasets. We analysed a total of 425 RNA-seq datasets (Table 1 and figshare^18^), consisting of 132 newly generated and 293 previously reported RNA-seq datasets, following the strategy outlined in Fig. 1. We further identified common AS genes related to environmental stressors, tissues, and signaling molecules. This approach will facilitate the investigation of AS using RNA-seq data collected from other related species. Furthermore, our findings may be applied to facilitate breeding programs focused on enhancing tolerance to environmental stressors in diverse crops, including those within the Solanaceae family.

**Table 1:** The overview of pepper transcriptomes used in this study.

Sample	Tissue/Treatment	Type of sample	Number of samples	Raw data (Gb)	GEO identifier
Tissues	Fruit development	placenta, pericarp	42	9.39	GSE240946^*^
	Tissue	root, stem, flower	8	7.50	
Environmental stressors	Biotic stress	oomycete, virus, bacteria	219	431.9	GSE240943^*^, GSE240944^*^, GSE240945^*^, GSE240946^*^, GSE240234
	Abiotic stress	cold, heat, osmosis, salinity	78	204.7	GSE240947^*^
Signaling molecules		SA, JA, ABA, ET	78	188	GSE240948^*^
Total			425	841.49	GSE240949 (SuperSeries) and GSE240234

^*^The SubSeries contained in SuperSeries GSE240949.

## Methods

### Inoculation and sample collection

*C. annuum* pepper seedlings were transferred to a 32-plug tray (6 cm in diameter and 6.5 cm in height) 2 weeks after germination and placed in a growth room at 24 ± 1 °C under a photoperiod with 16 h of light and 8 h of darkness. All plants were inoculated at the six true-leaf stage. For *Xanthomonas axonopodis* pv. *glycines* 8ra (Xag8ra), *Xanthomonas campestris* pv. *vesicatoria* race 1 (Xcv1), and *X. campestris* pv. *vesicatoria* race 3 (Xcv3), plants were injected with 10^8^ colony-forming units (cfu)/ml (optical density at 600 nm = 0.1), and the third or fourth leaves from four plants were harvested for RNA extraction at 0, 1, 3, 6, 12, and 24 h post-inoculation for Xag8ra and at 0, 3, 6, 12, 24, and 48 h post-inoculation for Xcv1 and Xcv3. Plants were inoculated with 5 × 10^4^ zoospore/ml of the oomycete *Phytophthora capsici* (*P. capsici*)^19^ and harvested at 0, 1, 2, 4, 6, 12, and 24 h post-infiltration. Tobacco mosaic virus P2 strain (TMV-P2) inoculum was prepared by homogenizing 1 g of infected leaves in 10 ml of 0.1 M phosphate buffer at pH 7.0^20^. Plants were then infected on the leaves by rubbing the inoculum with carborundum #400 (Hayashi Pure Chemical Ind., Japan). Following inoculation, the third or fourth leaves from the four plants were harvested for RNA extraction at 0, 0.5, 4, 24, 48, and 72 h. Three biological replicates were collected at each time point for each condition. Leaf samples were rapidly frozen in liquid nitrogen and stored at ‒80 °C until use.

### Library construction and RNA sequencing

Total RNA was isolated from 100-mg pepper leaf samples using TRIzol reagent (Ambion, USA), according to the manufacturer’s instructions. RNA was quantified using a NanoDrop 2000 spectrophotometer (Thermo Fisher Scientific, USA), and integrity was verified by agarose gel electrophoresis. A strand-specific library with inserts of approximately 150–200 bp in size was then constructed using 5 mg of each RNA sample, following a previously described method^21^, and a total of 132 cDNA libraries were generated for RNA-seq. The sequencing platforms and read lengths used for the different samples were as follows: *P. capsici*-infected plants were sequenced on the Illumina HiSeq 2500 platform (Illumina, USA) with 151-nt reads; Xcv1- and Xcv3-infected plants were sequenced on the HiSeq X Ten platform (Illumina) with 151-nt reads; Xag8ra-infected plants were sequenced on the HiSeq 2000 platform (Illumina) with 101-nt reads; and TMV-P2-infected plants were sequenced on the Illumina HiSeq 2500 platform with 101-nt reads. All of the 132 raw RNA-seq data were deposited to NCBI GEO with the SubSeries identifiers GSE240943 including 117 samples (39 *P. capsici*; 48 Xcv1,3; 30 Xag8ra) and 15 samples in GSE240944 (TMV-P2).

### Transcriptome dataset acquisition

A total of 293 RNA-seq datasets (490.79 Gb) were downloaded from NCBI and used for AS analysis. These include *C. annuum* samples from different tissues^11^ (50 samples are part of GSE240946) and those exposed to various environmental stressors^11,12^ (165 samples including 78 abiotic stress in GSE240947 and 87 biotic stress in GSE240945 and part of GSE240946) and signaling molecules^13^ (78 samples in GSE240948). A total of 425 RNA-seq datasets were used in this study (132 newly deposited and 293 downloaded RNA-seq datasets) and detailed information of samples is provided in Table 1 and figshare^18^.

### Construction of transcript model

The adapter sequences from all 425 RNA-seq datasets were removed from the raw RNA-seq reads using Cutadapt (v1.15)^22^, and low-quality reads with Phred score <20 were filtered out using Trimmomatic (v0.38)^23^, with the parameters “LEADING:3, TRAILING:3, SLIDINGWINDOW:4:20, and MINLEN:36”. After trimming, the quality of the trimmed reads was evaluated using FastQC^24^ and MultiQC^25^. Trimmed reads were then mapped to the *C. annuum* v.1.6 reference genome (http://peppergenome.snu.ac.kr)^10^ and the *C. annuum* 2.0 annotation gene model using HiSAT2 (v2.1.0)^26^ with default settings. Transcript assembly was performed using StringTie (v1.3.5) software^27^ with default parameters. After assembling the samples, an integrated transcript model was constructed using the merge function in StringTie. The reads counts were normalized using fragments per kilobase of transcript per million mapped reads (FPKM) for paired-end data and reads per kilobase of transcript per million mapped reads (RPKM) for single-end data. The normalized read count (GSE240234^28^) included a total of 219 samples, but only 132 newly generated samples (39 *P. capsici*, 48 Xcv1,3, 30 Xag8ra, 15 TMV-P2) were used for principal component analysis (PCA) for validation in this study. The remaining PCA was reported in the existing data descriptors^11–13^. Finally, in-house Perl scripts^18^ were used for transcript filtering to remove transcripts containing two or more annotated genes and exclude those without a stop codon (i.e., transcripts without a coding sequence).

### Analysis of AS and visualization

**Fig. 2:**
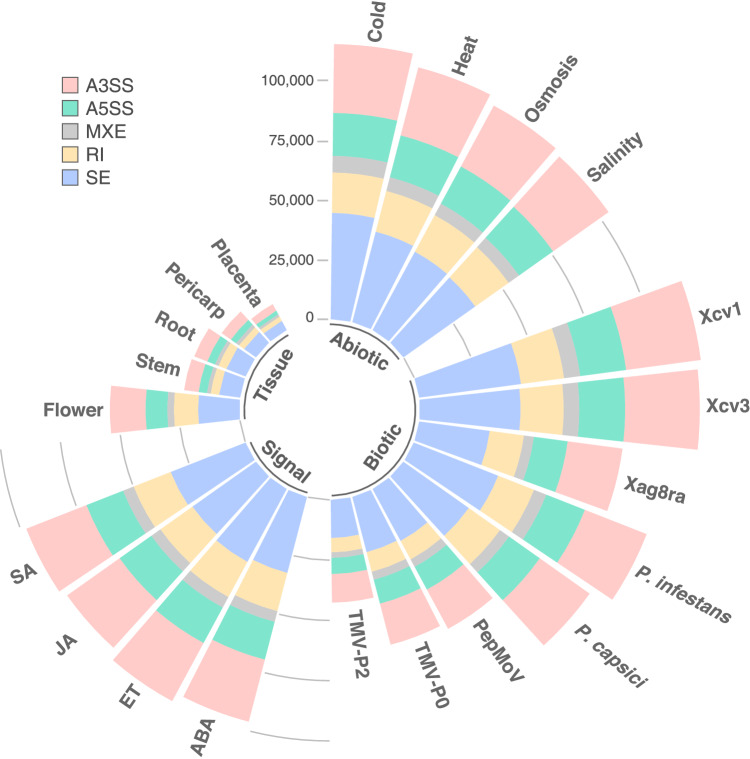
Overall patterns of alternative splicing (AS) distribution in *Capsicum annuum* transcriptome data collected from different tissues and in response to environmental stressors and signaling molecules. The circular stacked bar plot shows the number of AS events (pink-alternative 3′ splice site (A3SS), green-alternative 5′ splice site (A5SS), gray-mutually exclusive exons (MXE), yellow-intron retention (RI), blue-exon skipping (SE) induced by abiotic stressors, biotic stressors, signaling molecules, and in different tissues. Precise numbers of AS events in each group are shown in figshare^18^.

**Fig. 3:**
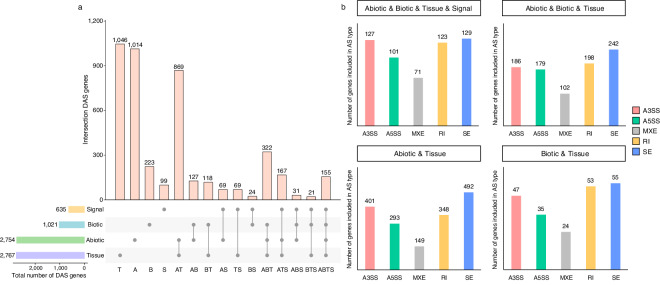
Overall patterns of differential alternative splicing (DAS) distribution under various conditions. The Benjamini–Hochberg method was applied to obtain p-values corrected for false-discovery rate (FDR), and DAS events were identified using a cutoff of |ΔIncLevel| ≥ 0.1 and FDR < 0.05. (**a**) UpSet plot depicts the number of specific and shared DAS genes across the four conditions signaling molecules (yellow), biotic stressors (blue), abiotic stressors (green), and tissues (purple). Each intersection set is represented by abbreviations below the intersection matrix: signaling molecules (S), biotic stressors (B), abiotic stressors (A), tissues (T). (**b**) Distribution of DAS gene AS types in response to abiotic stressors, biotic stressors, signaling molecules, and tissues intersection sets. The *x*-axis shows the five different AS types, and the *y*-axis indicates the number of DAS genes included for each AS type: pink-alternative 3′ splice site (A3SS), green-alternative 5′ splice site (A5SS), gray-mutually exclusive exons (MXE), yellow-intron retention (RI), blue-exon skipping (SE).

AS events were analyzed using rMATs (v4.0.2)^29^ based on the filtered gtf file generated by StringTie. AS patterns were classified into the following five different types of events: exon skipping (SE), intron retention (RI), mutually exclusive exons (MXE), alternative 3′ splice site (A3SS), and alternative 5′ splice site (A5SS), using the command “–cstat 0.0001 –libtype fr-firststrand”. We uncovered a total of 1,642,007 AS events. Biotic stressors resulted in the largest number of AS events (689,238), followed by abiotic stressors (433,339), signaling molecules (389,911), and tissues (129,519). In all conditions, SE was the most predominant type of alternative splicing, followed by A3SS, RI or A5SS (Fig. 2 and figshare^18^). Overall AS, specific AS and shared AS transcripts were counted using in-house Perl scripts^18^. Specific and shared AS transcripts were analyzed using the Benjamini–Hochberg method^30,31^, with a false-discovery rate (FDR) < 0.05, and |ΔIncLevel| ≥ 0.1 to identify differential alternative splicing (DAS). DAS genes refer to genes that undergo differential AS events in response to different stress conditions, resulting in the production of different isoforms compared to the control. DAS was quantified by rMATs using likelihood-ratio test to calculate the p-value and FDR to represent the inclusion level (IncLevel, also referred to as ψ) between stress samples and control. Detailed information about IncLevel and the algorithm are described in Shen *et al*.^29^. A total of 4,354 DAS genes^18^ were detected among all integrated datasets (Fig. 3). The highest number of specific DAS genes was identified in tissues (1,046), followed by abiotic stressors (1,014), biotic stressors (223), and signaling molecules (99). In addition, visualization of specific and shared AS transcripts was performed using the IGV browser^32^.

## Data Records

All RNA-seq data used in this study were deposited in SuperSeries GSE240949^33^. SuperSeries GSE240949^33^ comprises a total of six SubSeries (GSE240943-GSE240948), of which the 132 RNA-seq datasets newly generated in this study are contained in SubSeries GSE240943 (*P. capsici*, Xcv1, Xcv3, Xag8ra) and GSE240944 (TMV-P2), respectively. The remaining previously reported RNA-seq data were downloaded from the GEO IDs in Table 1. Overview of transcriptomes, quality assessment of 132 RNA-seq datasets, PCA, information of AS events generated in the course of this study, and used code have been deposited in figshare^18^.

## Technical Validation

### Quality control

Quality assessment of the new generated 132 RNA-seq dataset was performed using FastQC and is summarized in a report applying MultiQC. The sequencing results were of high quality indicated by a mean Phred score above 20 per sequence (figshare^18^ File 2 a-e) and a mean Phred score above 25 per read (figshare^18^ File 2 f-j). The mapping rates of the trimmed reads including all datasets, which average 88.19% in the *C. annuum* v.1.6 reference genome, have been deposited to figshare^18^ with statistical summaries. The raw reads and normalized read counts have been submitted to NCBI GEO under subseries GSE240943 and GSE240944 for raw reads and GSE240234^28^ for normalized read counts. To assess the variation between samples, principal component analysis (PCA) was performed for each stressor including *P. capsici*, TMV-P2, Xag8ra, Xcv1, and Xcv3^18^. The results showed that mock and pathogen were separately grouped in each stressor.

### Alternative splicing patterns in 425 RNA-seq datasets

**Fig. 4:**
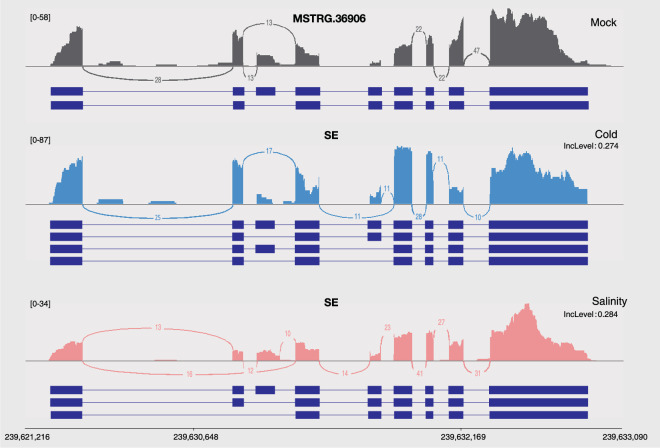
Visualization of alternative splicing isoforms of differential alternative splicing (DAS) gene MSTRG.36906 under cold and salinity stress. Sashimi plots depict differential alternative splicing (DAS) through exon skipping (SE) events of gene MSTRG.36906 in response to different abiotic stressors. Individual plots for control (mock, gray), cold stress (blue), and salinity stress (pink) visualize the read coverage and junction reads which are plotted as arcs with the indicated junction read counts. Below each plot alternative splicing isoforms are visualized in dark blue. The Benjamini–Hochberg method was applied to obtain p-values corrected for false-discovery rate (FDR), and DAS events were identified using a cutoff of |ΔIncLevel| ≥ 0.1 and FDR < 0.05.

**Fig. 5:**
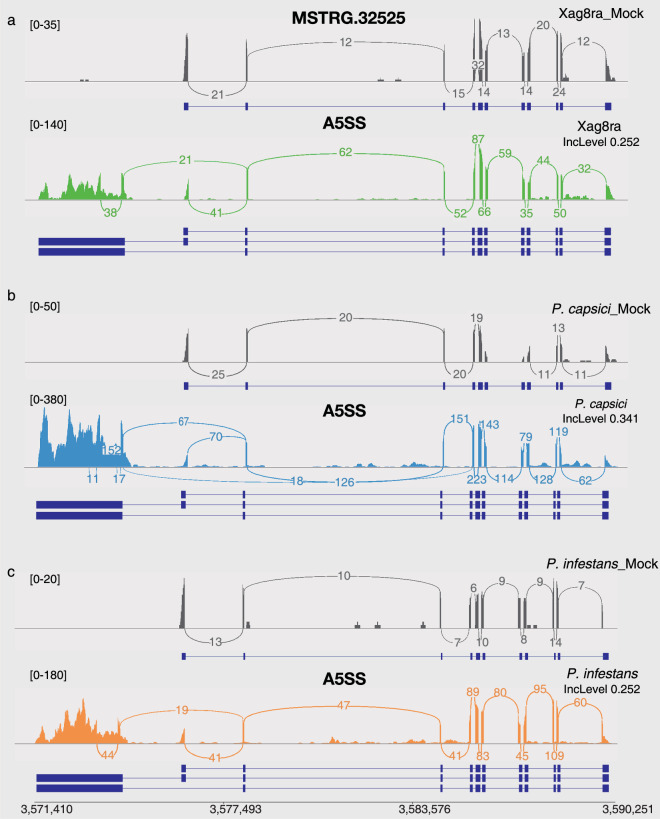
The Sashimi plot of DAS gene MSTRG.32525 undergo differential alternative splicing under biotic stressors. Sashimi plots depict differential alternative splicing (DAS) through alternative 5′ splice site (A5SS) events of gene MSTRG.32525 in response to different biotic stressors. Individual plots visualize the read coverage and junction reads which are plotted as arcs with the indicated junction read counts. Below each plot alternative splicing isoforms are visualized in dark blue. (**a**) Control (Xag8ra_mock, gray) and AS in response to *Xanthomonas axonopodis* pv. *glycine* 8ra (Xag8ra, green), (**b**) control (*P. capsici*_mock, gray) and AS in response to *Phytophthora capsici* (*P. capsici*, blue), (**c**) control (*P. infestans*_mock, gray) and AS in response to *Phytophthora infestans* (*P. infestans*, orange). The Benjamini–Hochberg method was applied to obtain p-values corrected for false-discovery rate (FDR), and DAS events were identified using a cutoff of |ΔIncLevel| ≥ 0.1 and FDR < 0.05.

To validate AS patterns in the RNA-seq datasets, we calculated the number of DAS genes that were shared and variable across the different stress conditions (Fig. 3a). There were a total of 155 shared DAS genes that underwent AS in the ABTS intersection set. When listing the number of shared DAS genes among any of these groups in descending order, excluding those shared in the ABTS intersection set, the largest four groups are as follows: 869 in the AT intersection set, 322 in the ABT intersection set, 167 in the ATS intersection set, and 127 in the AB intersection set. Next, we compared the DAS genes identified under abiotic and biotic stressors to the tissues with the highest number of DAS genes (1,046) to identify variable DAS gene AS types in the ABTS, ABT, AT and BT intersection sets (Fig. 3b). To confirm the quality of alternative splicing isoforms in response to stress, we randomly selected two DAS genes of the 4,354 DAS genes for visualization. Under cold and salinity stress, the DAS gene MSTRG.36906, an XRI1-like protein related with the DNA repair and cell division^34^, underwent differential alternative splicing through SE events as shown in Fig. 4. Similarly, MSTRG.32525 gene, a VPS2.3-like protein required for membrane remodeling events^35^, was also differentially alternative spliced through A5SS events when exposed to Xag8ra, *P. capsici*, and *P. infestans* (Fig. 5).

## Usage Notes

We generated and submitted 132 novel RNA-seq datasets, including the oomycete *P. capsici*, the tobacco mosaic virus TMV-P0, and three different bacterial species, namely Xag8ra, Xcv1, and Xcv3, to NCBI GEO (GSE240943, GSE240944). The present study provides information about RNA-seq datasets that can be used to examine expression profiles, analyze AS, and investigate further research related to environmental stressors.

We propose an approach to analyze differential AS from massive RNA-seq datasets. This approach may further also be useful for investigating unknown common factors associated with various stressors or conditions. Additionally, our findings provide useful data for basic-research programs focused on enhancing tolerance to environmental stressors in hot pepper or establishing breeding programs.

## Data Availability

The code used in this study is deposited to figshare (10.6084/m9.figshare.23671647.v7)^18^.
